# Management of Surgical Aortic Valve Replacement Degeneration With Transcatheter Aortic Valve Implantation (TAVI in SAVR): Experience in Nicaragua

**DOI:** 10.7759/cureus.76195

**Published:** 2024-12-22

**Authors:** Juan Cristobal Mendoza, Álvaro Morales, Mario Monjarrez Vega, Christopher Romero, Gery Castrillo Borge, Cesar Baltodano Dangla

**Affiliations:** 1 Cardiology, Hospital Militar Escuela "Dr. Alejandro Dávila Bolaños", Managua, NIC; 2 School of Medicine, Hospital Militar Escuela "Dr. Alejandro Dávila Bolaños", Managua, NIC

**Keywords:** aortic stenosis (as), dyspnea, low-flow low-gradient severe aortic stenosis, surgical aortic valve replacement (savr), trans catheter aortic valve implantation (tavi)

## Abstract

Severe aortic valve stenosis poses a significant risk for the aging population, often escalating from mild symptoms to life-threatening heart failure and sudden death. Without timely intervention, this condition can lead to disastrous outcomes. The advent of transcatheter aortic valve implantation (TAVI) has gained popularity, emerging as an effective alternative for managing severe aortic stenosis (AS) in high-risk patients experiencing deterioration of previously implanted bioprosthetic surgical aortic valves (SAV), which introduces complex challenges such as device compatibility and anatomical considerations. We report the case of a 76-year-old male with a history of stage III hypertension, compensated type 2 diabetes, and aortic valve disease who underwent bioprosthetic valve replacement in 2013. His medications included metoprolol, metformin/glibenclamide, and levothyroxine. He presented with moderate exertional dyspnea (NYHA II) over four months, relieved by rest. Physical examination revealed a crescendo-decrescendo systolic murmur at the aortic focus. The aortic prosthesis stenosis was confirmed by a transthoracic echocardiogram. A CT angiogram showed bioprosthetic degeneration and significant calcification, allowing for transcatheter aortic valve implantation in the surgical aortic valve. The procedure was successfully performed via the transfemoral route using a 21.5 mm MyVal balloon-expandable valve. The intervention improved the patient’s quality of life, resolving NYHA class III dyspnea and enabling greater independence in daily activities. Echocardiographic findings demonstrated a significant reduction in the transvalvular gradient, enhancing cardiac function and eliminating the immediate risk of valvular dysfunction progression, contributing to increased life expectancy and emotional well-being. This case highlights the feasibility and clinical benefits of transcatheter aortic valve implantation in surgical aortic valves for managing valve degeneration in a resource-limited setting, thereby representing a significant advancement in the treatment of aortic valve disease. The successful outcome demonstrates the importance of adopting innovative, minimally invasive techniques, particularly in regions with limited advanced interventions, by alleviating dyspnea, enhancing cardiac function, and significantly improving the patient’s quality of life, emotional well-being, and prognosis.

## Introduction

Severe symptomatic aortic stenosis (AS) carries a mortality risk of 50% in the first year if appropriate intervention is not performed [1]. Since the introduction of TAVI, this technique has gained popularity, surpassing surgical replacement, with over 200,000 procedures performed annually worldwide [2]. Transcatheter aortic valve implantation in surgical valve (TAVI in SAV) has established itself as an effective alternative for managing severe aortic stenosis in high-risk patients who experience structural or functional deterioration of previously implanted bioprosthetic valves. This procedure has become essential, considering that up to 20% of patients receiving surgical aortic valve replacement present with stenosis or valvular insufficiency within a five-year period [3]. Transcatheter aortic valve replacement (TAVR) in SAV presents a viable solution; this approach is less invasive than redo surgical valve replacement and is increasingly offered to patients at lower surgical risk. However, this procedure entails a series of challenges, such as the risk of elevated post-procedural gradients, coronary obstruction, and calcification in the left ventricular outflow tract or the aortic annulus, including the need for personalized planning that considers the anatomical particularities of each patient and the interaction of the devices with the coronary arteries [3].

## Case presentation

A 76-year-old patient with a history of stage III hypertension, compensated type 2 diabetes with a body mass index of 26.1, and stage D1 aortic valvular heart disease underwent valve replacement with a 21 mm bioprosthetic valve, Edwards model 3300TFX, in 2013. Currently, he is being treated with metoprolol 25 mg (1/2 tablet daily), metformin with glibenclamide 500/5 mg (one tablet daily), and levothyroxine 0.25 mcg (one tablet daily). The patient reports no alcohol, tobacco, or drug use.

He has a clinical history of moderate-intensity exertional dyspnea, NYHA class II, for approximately four months, which improves with rest. Upon admission, the chest was symmetrical and expandable, with no displacement of the apex beat. Blood pressure was 113/74 mmHg, with a mean arterial pressure of 88 mmHg and a heart rate of 76 bpm. Cardiac auscultation revealed a regular rhythm with the presence of a crescendo-decrescendo ejective systolic murmur at the aortic focus and an accessory aortic focus with intensity 3/6, radiating to the neck arteries.

A transthoracic echocardiogram revealed that the Edwards 3300TFX prosthetic valve in the aortic position had stenosis (maximum velocity: 2.2 m/s, maximum gradient: 30.7 mmHg, mean gradient: 16.1 mmHg, valve area: 2.65 cm²). Blood tests, including hematological, electrolyte, and renal function tests, showed the following results (Table 1). The patient presented with mild anemia, with a hemoglobin level consistent with grade I anemia according to the WHO classification. While this mild anemia is unlikely to be the sole cause of his symptoms, it may contribute to the overall symptom burden.

**Table 1:** Laboratory tests

Laboratory tests	Results	Reference range
Hemoglobin	11.5 g/dL	13–17 g/dL
Hematocrit	34.6%	39–50%
White blood cell count	6.22³/μL	5–10 x 10³/µL
Platelets	185³/μL	150–500 10³/µL
Neutrophils (%)	53.8%	55–65%
Lymphocytes	31.8%	25–35%
Creatinine	0.9 mg/dL	<1.1 mg/dL
Serum calcium	9.29 mg/dL	8.4–9.6 mg/dL
Potassium	4.2 mmol/L	3.5–5.1 mmol/L
Magnesium	2.1 mg/dL	1.63–2.41 mmol/L
Sodium	141.6 mmol/L	136–145 mmol/L
Partial thromboplastin time	25 sec	22.7–32.5 sec
Prothrombin time	11.2 sec	9.6–12 sec
International normalized ratio	1.08	0.8–1.1
Procalcitonin	0.04 ng/dL	<0.5 ng/dL
Polymerase chain reaction (PCR)	0.24 mg/dL	<0.5 mg/dL

**Figure 1:**
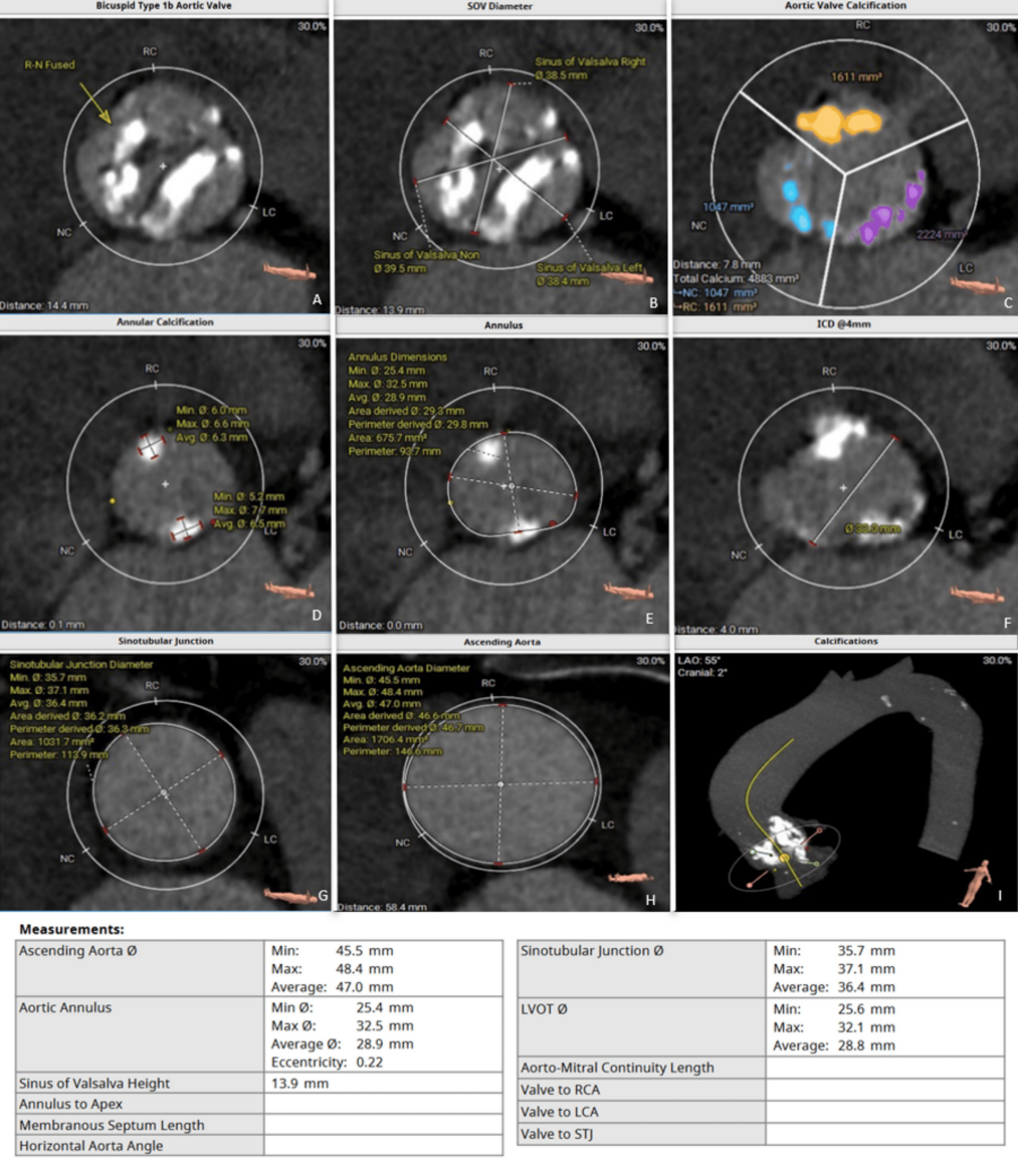
(A-L) Computed tomography angiography for the transcatheter aortic valve replacement protocol The findings reveal a bicuspid aortic valve type 1b, with severe calcification on the leaflets. These calcifications extend from the right coronary cusp and the left coronary cusp to the left ventricular outflow tract, significantly affecting the valvular anatomy. Additionally, the dimensions of the left ventricular outflow tract are comparable to those of the aortic annulus. A mild dilation of the ascending aorta is also noted, presenting some calcified atherosclerotic plaques. The aortic arch is displaced to the left and also shows the presence of atherosclerotic plaques. Finally, the supra-aortic trunks present atherosclerotic plaques, but no evidence of significant stenosis is found in the evaluated areas.

**Figure 2:**
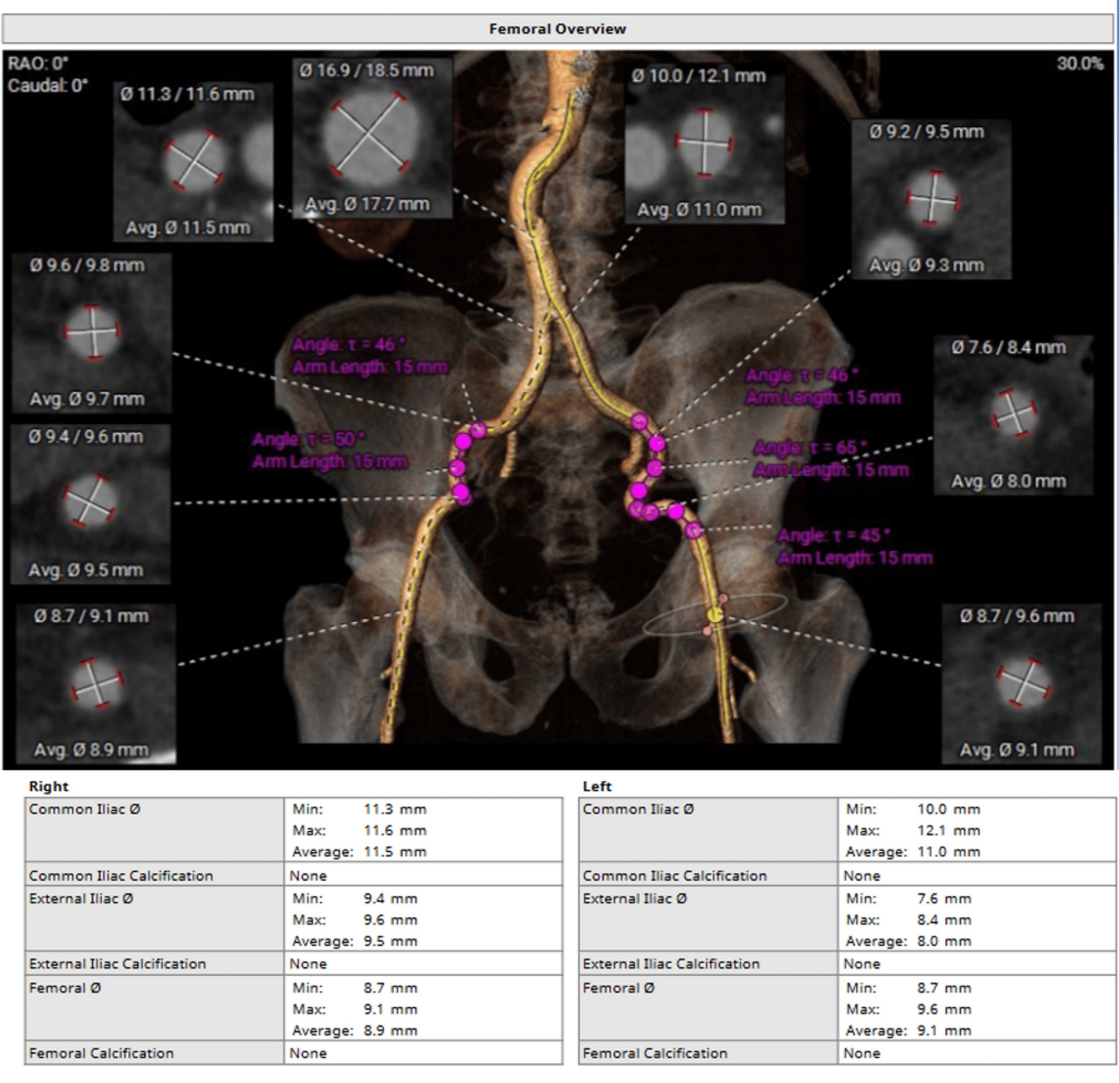
Femoral overview from computed tomography angiography for TAVR The abdominal aorta showed an adequate caliber, with no evidence of aneurysmal dilation or significant atherosclerotic plaques. The iliac and femoral arteries presented sufficient diameter for device insertion, with minimal calcifications and no relevant stenosis.

In the angiotomography performed on the patient, degeneration of the previously implanted bioprosthesis was evidenced, showing severe stenosis and significant calcification (Figure 1A-1F), and being at high risk for surgical reoperation, meeting the criteria, it was decided to perform transcatheter aortic valve implantation in the surgical aortic valve (valve-in-valve TAVI). Furthermore, it was confirmed that the valve annulus and the aortic root have an adequate diameter for the placement of a new valve, with no risk of coronary obstruction and favorable vascular access, given the caliber and condition of the femoral and iliac arteries (Figure 2). Since all required anatomical and structural parameters are met, the decision is made to schedule two months later and initiate the transcatheter aortic valve replacement procedure promptly.

**Figure 3:**
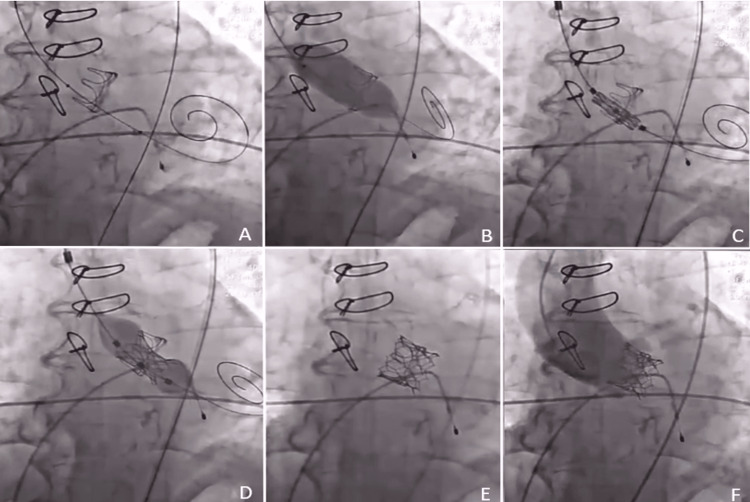
Transcatheter aortic valve implantation in surgical aortic valve procedure Through the right femoral artery, a 6 Fr diagnostic AL 2 catheter was introduced, and with a straight hydrophilic guide, it was advanced to the left ventricle. The AL catheter was exchanged for an INNOVE-EZ 0.035” × 270 cm guide, and the AL catheter was removed. The SAMVALVE balloon 22 mm × 40 mm was advanced to the valvular ring, where pacing was done at 180 beats per minute, and the balloon was inflated manually until the waist ruptured, observing no electrocardiographic changes. The balloon was removed, and the valve mounted on the MyVal balloon (21.5 mm) was advanced and subsequently inflated without complications, with control performed using contrast injection, showing the valve in an adequate position without paravalvular leak.

A TAVI in SAV was successfully performed using a balloon-expandable MyVal valve of 21.5 mm and a CoreValve Evolut of 23 mm. Additionally, two BENTLY stents, measuring 8 mm × 27 mm and 9 mm × 57 mm, respectively, were placed in the right common femoral artery (Figure 3).

A TAVI IN SAV was successfully performed using the MyVal 21.5 mm balloon-expandable valve, and two BENTLY stents, 8 mm × 27 mm and 9 mm × 57 mm, were placed in the right common femoral artery.

The patient experienced no procedural complications during or after the TAVR procedure. The post-procedural recovery was uneventful, with no issues at the femoral puncture sites, no development of AV block, no tachyarrhythmias, and no hemodynamic disturbances. The patient was discharged approximately 36 hours following the procedure.

The follow-up protocol involved a one-month follow-up appointment, during which an echocardiogram was performed to assess the function of the prosthetic aortic valve. In the meantime, the patient was instructed to rest for one week, avoid repetitive hip flexion movements, and monitor for pain or bleeding at the femoral puncture sites. A follow-up cardiology outpatient appointment was also scheduled for one month later to ensure proper recovery and to manage any potential issues.

The patient underwent an echocardiogram evaluation at the one-month follow-up, revealing favorable results. Key findings included a jet velocity of less than 3 m/s, a left ventricular outflow tract of 22 cm, a velocity-time integral (VTI) in the aortic valve of 57 cm, a left ventricular outflow tract diameter of 1.67 cm, and an acceleration time of 97 ms. These findings confirmed an improvement in the patient's condition, supporting the success of the procedure and the effectiveness of the follow-up protocol.

With the intervention, the patient progressed from experiencing NYHA class II dyspnea to tolerating long walks without difficulty, significantly improving their independence in daily activities. Additionally, the immediate risk of progression of valvular dysfunction was eliminated, providing the patient with a greater life expectancy and improved emotional well-being.

## Discussion

Clinically significant AS affects approximately 1.6% of adults over the age of 65 [4]. In a study involving patients with severe aortic stenosis, those who received only medical treatment exhibited an overall mortality rate of 81% during a mean follow-up of 3.9 years, compared to 43% for those who underwent TAVI [5]. This highlights the poor prognosis associated with conservative management in severe aortic stenosis.

The first documented case of TAVI performed within a previously implanted surgical aortic valve, utilizing the transaortic approach, was reported by Cockburn et al. [6]. Since then, a series of cases have been reported, with the terminology evolving and varying within the literature. Terms such as "ViV TAVI" refer to a transcatheter valve placed within a previously implanted surgical bioprosthesis due to structural deterioration of the valve. This definition is crucial for distinguishing ViV procedures from other transcatheter interventions, such as redo-TAVI, which refers to the procedure of implanting a new transcatheter aortic valve within a previously placed transcatheter heart valve that has failed [7].

The primary indications for this procedure are mainly for patients with symptomatic degeneration of a surgical bioprosthetic aortic valve who are at high or prohibitive risk for reoperation. Degeneration may present as stenosis, regurgitation, or a combination of both [7].

Diabetes accelerates the deterioration of bioprosthetic valves primarily through increased calcification and inflammation. Hyperglycemia in diabetic patients enhances oxidative stress and activates key pro-inflammatory pathways. In particular, the expression of nuclear factor kappa B (NF-κB) and bone morphogenetic protein 2 (BMP-2) is significantly increased in individuals with aortic stenosis, favoring both inflammation and valvular calcification [8]. This inflammatory environment is also characterized by an increased expression of chondro-osteogenic markers, such as Runx2 and alkaline phosphatase (ALP), which are essential in the calcification process [9].

Furthermore, diabetes induces endothelial dysfunction in valvular endothelial cells (VEC) and valvular interstitial cells (VIC), leading to tissue remodeling changes that promote calcification [10]. High glucose conditions stimulate the production of cytokines, cell adhesion molecules, and matrix metalloproteinases (MMP), contributing to a favorable environment for calcification. These effects are supported by studies showing that elevated glucose concentrations activate BMP and TGF-β signaling pathways, further enhancing osteogenic differentiation and calcification [10].

Hypertension exacerbates these processes by increasing mechanical stress on the bioprosthetic valve. Elevated blood pressure levels intensify shear stress, velocity, and vorticity near the leaflets, which may initiate or accelerate calcific changes [11]. This hemodynamic stress, combined with the inflammatory and pro-calcific environment induced by diabetes, results in more rapid deterioration of the bioprosthetic valve, such as in our patients.

Meticulous planning through angiography is essential for evaluating the anatomy of the degenerated valve, the aortic root, and the coronary ostia, as well as the presence of calcifications, allowing for the selection of the appropriate size and type of transcatheter valve. Smaller surgical bioprostheses (≤21 mm) present higher risks of residual gradients and prosthesis-patient mismatch, translating into lower hemodynamic efficiency after a ViV [12,13].

The MyVal valve, measuring 21.5 mm, offers a slight increase in the effective orifice area, reducing gradients. The favorable outcomes of the therapeutic process are associated not only with the technique but also with the type of valve. The MyVal valve has demonstrated high technical success rates and favorable short- and mid-term results. For instance, a multicenter study reported a technical success rate of 98% and an overall survival rate of 92% at 15 months in patients undergoing ViV or valve-in-ring (ViR) implantation with the MyVal valve; that is the reason it was used in this patient; however, each patient must be treated individually [14].

Regarding complications, a meta-analysis including 5,553 patients reported a procedural success rate of 97%. At 30 days, all-cause mortality was 5%, stroke occurred in 2%, myocardial infarction in 1%, and 6% required permanent pacemaker placement. At one year, all-cause mortality increased to 12%, 3% of patients experienced a stroke, 1% presented with myocardial infarction, and 7% needed a permanent pacemaker [15]. Additionally, improper initial positioning of the transcatheter valve may occur, necessitating repositioning or placement of a second valve, and elevated transvalvular gradients after the procedure can lead to suboptimal hemodynamic outcomes and increased long-term mortality. Achieving a mean gradient of less than 20 mmHg is associated with better outcomes [16].

Elevated gradients across the aortic valve increase afterload, leading to left ventricular hypertrophy, higher myocardial oxygen demand, reduced coronary reserve, and increased risk of ischemia, heart failure, and mortality. When the prosthetic valve's effective orifice area is too small, it causes high-pressure gradients, limiting LV hypertrophy regression and contributing to heart failure, faster valve degeneration, and reduced survival. Hemodynamic valve deterioration (HVD), marked by increased gradients or regurgitation, worsens LV function and remodeling, leading to poor survival outcomes. High transprosthetic gradients can also trigger arrhythmias and limit exercise capacity, which negatively affect functional status and quality of life, increasing mortality risk [17,18].

## Conclusions

This case emphasizes the critical importance of advancing and integrating cutting-edge techniques in interventional cardiology, showcasing how high-quality care can be delivered even in the most resource-challenged environments, such as developing nations. The successful execution of transcatheter aortic valve implantation within a surgical aortic valve not only proves to be feasible but offers significant therapeutic benefits. It grants access to revolutionary treatments that could revolutionize the approach to managing aortic valve disease in the region. Particularly within this context, where such cases have never been reported, this example sets a compelling precedent that could inspire neighboring countries to explore similar transformative solutions.

The ViV technique offers an elegant, minimally invasive alternative to traditional open-heart surgery, with a marked reduction in both short- and long-term complications. More than just a technical triumph, this approach enhances the patient's overall well-being dramatically, alleviating symptoms, improving cardiac function, and providing a notable boost to mental health, all of which collectively contribute to a substantially improved prognosis. The resounding success of this case reinforces the need for continued exploration and adoption of minimally invasive methods, particularly in settings where resources are limited. It also highlights the immense potential for these procedures to unlock new pathways to healthcare excellence, enabling the delivery of life-saving treatments and improved quality of life to populations who would otherwise have limited access to such advanced interventions. This landmark achievement serves not only as a success but also as a catalyst for future progress in the region’s healthcare landscape.
